# Large Amplitude Motions of Pyruvic Acid (CH_3_-CO-COOH)

**DOI:** 10.3390/molecules26144269

**Published:** 2021-07-14

**Authors:** María Luisa Senent, Samira Dalbouha

**Affiliations:** 1 Theoretical Chemistry and Physics Department Institute for the Structure of Matter IEM-CSIC, Serrano 121, 28006 Madrid, Spain; 2 Laboratory of Applied Chemistry and Environment, Department of Chemistry, Faculty of Science, Ibn Zohr University, Agadir B.P. 8106, Morocco; samiradalbouha@gmail.com; 3 Laboratory of Spectroscopy, Molecular Modeling, Materials, Nanomaterials, Water and Environment, Department of Chemistry, Faculty of Sciences, University Mohammed V, Avenue Ibn Battouta, Rabat B.P. 1014, Morocco

**Keywords:** pyruvic acid, LAM, VOC, torsion, spectrum, atmosphere

## Abstract

Torsional and rotational spectroscopic properties of pyruvic acid are determined using highly correlated ab initio methods and combining two different theoretical approaches: Second order perturbation theory and a variational procedure in three-dimensions. Four equilibrium geometries of pyruvic acid, Tc, Tt, Ct, and CC, outcome from a search with CCSD(T)-F12. All of them can be classified in the C_s_ point group. The variational calculations are performed considering the three internal rotation modes responsible for the non-rigidity as independent coordinates. More than 50 torsional energy levels (including torsional subcomponents) are localized in the 406–986 cm^−1^ region and represent excitations of the ν_24_ (skeletal torsion) and the ν_23_ (methyl torsion) modes. The third independent variable, the OH torsion, interacts strongly with ν_23_. The A_1_/E splitting of the ground vibrational state has been evaluated to be 0.024 cm^−1^ as it was expected given the high of the methyl torsional barrier (338 cm^−1^). A very good agreement with respect to previous experimental data concerning fundamental frequencies (ν^CAL^ − ν^EXP^ ~ 1 cm^−1^), and rotational parameters (B_0_^CAL^ − B_0_^EXP^ < 5 MHz), is obtained.

## 1. Introduction

Pyruvic acid (CH_3_CO-COOH, PA) is a prevalent species in the Earth’s atmosphere [1,2] where it is a key intermediate of keto-acid reactions. Its presence has been reported in gas phase, aerosols, fogs, clouds, polar ice, and rainwater. Primary sources of pyruvic acid in the atmosphere are the reaction between the hydroxyl radical and hydrated methylgloxal, the oxidation of isoprene, and the ozonolysis of methyl vinyl ketone [3]. In regions with abundant vegetation, it can be emitted directly by plants. The presence of PA can alter the chemical and optical properties of atmospheric particles. It acts as a key intermediate in the oxidative channels of isoprene and secondary organic aerosols (SOA) formation.

Photo-oxidation of volatile organic compounds (VOC) yields products that are precursors of SOAs. The photodissociation of pyruvic acid displays a complex and significant chemistry in the atmosphere [1,2]. It absorbs light in the near-UV and by photodissociation, it decomposes forming CO_2_, additional products, and a reactive intermediate, methylhydroxycarbene, which produces acetaldehyde by isomerization. The photolysis represents the main destructor of the acid since it oxidized relatively slowly by peroxides and OxH radicals [2]. Vaida et al., who have published a long list of recent papers focused on PA [4,5,6] and references therein], have explored the multiphase photochemistry to elucidate its likely primary atmospheric removal pathways and the implications for SOA. Some of these works attend to the role of the conformers and the spectroscopic properties of the acid on its reactivity. Since the high overtone excitation of the OH-stretching mode in the gas phase leads to a unimolecular decarboxylation reaction, the dynamics of overtone-excited pyruvic acid (PA) was the object of a meticulous study using a combination of theoretical methods and measurements of the OH-stretch fundamental and overtone transitions with Fourier transform infrared spectrometry [7,8].

In addition to the earth atmosphere relevance, carboxylic acids are present in the volatile fraction of carbonaceous meteorites [9]. Examples are compounds such as acetic acid already observed in extraterrestrial atmospheres in the gas phase [10]. Recently, Kleimeier et al. [11] have designated pyruvic acid to be a detectable species in extraterrestrial sources. They have studied its formation by barrierless recombination of hydroxycarbonyl (HOCO) and acetyl (CH_3_CO) radicals in ices of acetaldehyde (CH_3_CHO) and carbon dioxide (CO_2_) modeling interstellar conditions driven by cosmic rays.

Motivated by a possible search of PA in gas phase sources, Kisiel et al. [12] measured the millimeter wave rotational spectrum in the region at 160–314 GHz and in supersonic expansion at 10–17.4 GHz. They provide parameters for various torsional states initiating the analysis on the basis of previous work predictions [13,14,15,16]. Dyllic-Brenzinger et al. [15] observed an anomalous behavior in the first excited vibrational state in the rotational spectrum that they attributed to an interaction between the two low-lying torsional modes. Meyer and Bauder [16] used a two-dimensional flexible model to predict the energies of the lowest vibrational states and the A/E splittings in the second exited ν_24_ torsional state. The barrier to internal rotation was established to be V_3_ = 965 ± 40 cal/mol. The stark effect measurements of Marstokk and Möllendal [14] yielded to μ_a_ = 2.27 ± 0.02 D, μ_b_ = 0.35 ± 0.02 D, and μ = 2.3 ± 0.03 D.

The vibrational spectrum was measured in the gas phase and in Ar matrix by Holleisntein et al. in 1978 [17]. More recently, the vibrational spectra of the two most stable conformers were measured in N_2_ and Ar matrices and analyzed by Reva et al. [18]. Ab initio calculations have been performed to search for stable geometries [19]. PA was studied as a benchmark to test theoretical studies of conformers of flexible molecules performed combining couple cluster (CC) and DFT methodologies [20].

In this paper, we attend to the pyruvic acid from the point of view of ab initio calculations with special attention to the far infrared region (FIR). The work describes structural and spectroscopic properties following the procedures used for molecules such as acetic acid [21], acetone [22] or biacetyl [23], which are properties or functional groups with PA. Ab initio calculations are achieved using explicitly correlated coupled cluster theory [24,25]. The far infrared region, relevant for rotational studies, is explored using a three-dimensional variational procedure [26]. Recently, this procedure has been improved and implemented for a proper classification of the energy levels in molecules with regions showing a high density of states [27]. In the procedure, the two low-lying torsional modes are considered to be independent coordinates. These two vibrational modes are strongly coupled as was early observed [15]. A third coordinate, the OH internal rotation, is defined as an independent coordinate since it interacts strongly with the ν_24_ mode.

## 2. Results

### 2.1. Electronic Structure Calculations

The geometrical parameters and energies of the pyruvic acid conformers were computed using the explicitly correlated coupled cluster theory with singles and doubles substitutions augmented by a perturbative treatment of triple excitations (CCSD(T)-F12 [24,25] as it is implemented in MOLPRO [28], using the default options and the AVTZ-F12 basis set. This basis set contains the aug-cc-pVTZ (AVTZ) [29] atomic orbitals and the default functions for the density fitting and the resolutions of the identity. To obtain accurate rotational constants, the core-valence electron correlation effects on the structures were introduced using CCSD(T) [30] and the cc-pCVTZ basis set (CVTZ) [31].

Two different theoretical models were applied for determining the spectroscopic parameters: The vibrational second order perturbation theory (VPT2) [32] implemented in Gaussian [33], and a variational procedure of reduced dimensionality implemented in our code ENEDIM [34,35,36]. If VPT2 is applied, the four conformers are treated as four different semi-rigid species, unable to interconvert, and the vibrations are described as small displacements around the equilibrium geometries. However, if the variational procedure is employed, the non-rigidity is taken into account. The four minima are treated together and their interconversion is considered implicitly.

For VPT2, the quadratic, cubic, and quartic terms of a force field were computed using the second order Möller-Plesset theory (MP2) and the AVTZ basis set [29]. For the variational calculations a three-dimensional potential energy surface was obtained at the CCSD(T)-F12/AVTZ-F12 level of theory applied on a grid of structures partially optimized with MP2/AVTZ. The surface was vibrationally corrected using MP2/AVTZ harmonic calculations.

### 2.2. The Four Conformers of Pyruvic Acid

**Figure 1:**
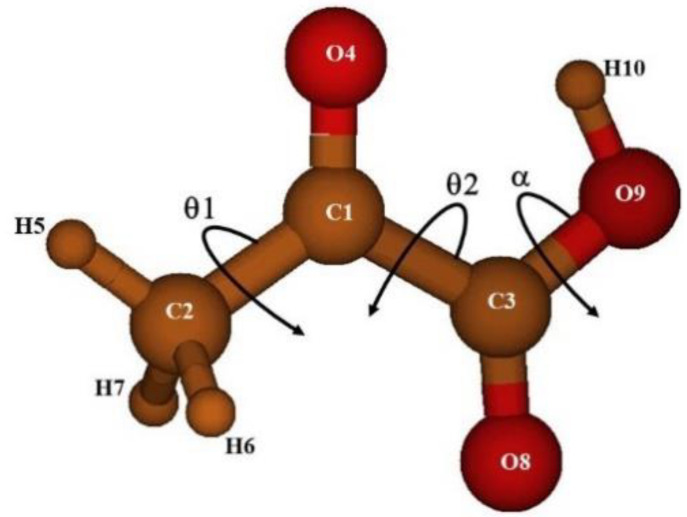
The preferred geometry of pyruvic acid.

Four equilibrium geometries of pyruvic acid, Tc, Tt, Ct, and CC have been identified based on a search with CCSD(T)-F12. All of them present a symmetry plane and can be classified in the C_s_ point group. The most stable geometry is the Tc (TRANS-cis) conformer represented in Figure 1. “TRANS and CIS” designate the relative orientation of the two C=O groups, whereas “trans and cis” denote the relative position of the C1 and H10 atoms.

Table 1 summarizes the properties characterizing the four conformers: The CCSD(T)-F12/AVTZ-F12 relative energies E, and the corresponding vibrationally corrected relative energies, E^ZPVE^; the CCSD(T)-F12/AVTZ-F12 equilibrium rotational constants, the MP2/AVTZ dipole moment components along the principal axis, the high of the barriers restricting the minimum interconversion, and the three coordinates, θ_1_, θ_2_, and α, that identify the structures. They correspond to the methyl group torsion, the torsion of the C3-C1 bond, and the hydroxyl group torsion, respectively. In the variational procedure described in the next sections, θ_1_, θ_2_, and α represent the independent coordinates. They are defined as the following linear combination of internal coordinates:

θ_1_ = (H5C2C1C3 + H6C2C1C3 + H7C2C1C3)/3 θ_2_ = O8C2C1C3 + O9C2C1C3 − 180· α = H10O9C3O8(1)

In Table 1, the computed dipole moment components are compared with previous experimental data derived from stark effect measurements [14].

**Figure 2:**
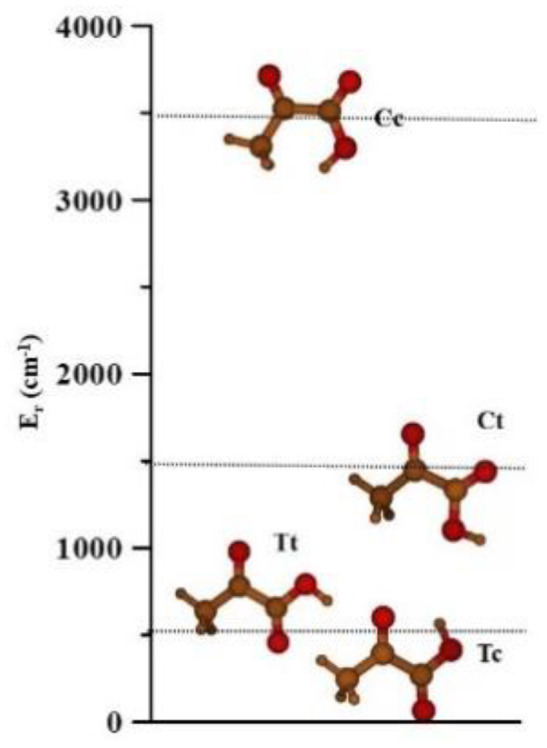
The four pyruvic acid conformers. The relative energies are given in Table 1.

Figure 2 represents the relative stabilities of the four conformers. The CCSD(T)-F12/AVTZ-F12 structural parameters and MP2/AVTZ anharmonic vibrational frequencies are collected in Table 2 and Table 3, respectively.

The Tc form stabilizes by the formation of an intramolecular hydrogen bond (H10….O4 = 2.0030 Å). The next two conformers Tt and Ct lie in the 950–2000 cm^−1^ region. In previous papers [18], the higher energy conformer Cc is ignored since it is very unlikely to be observed. Although, it is described as a transition state by several levels of theory, all the MP2/AVTZ harmonic wavenumbers are real. The low stability of Cc is due to the non-bonding repulsions between H10 and its neighboring methyl group hydrogens.

**Figure 3:**
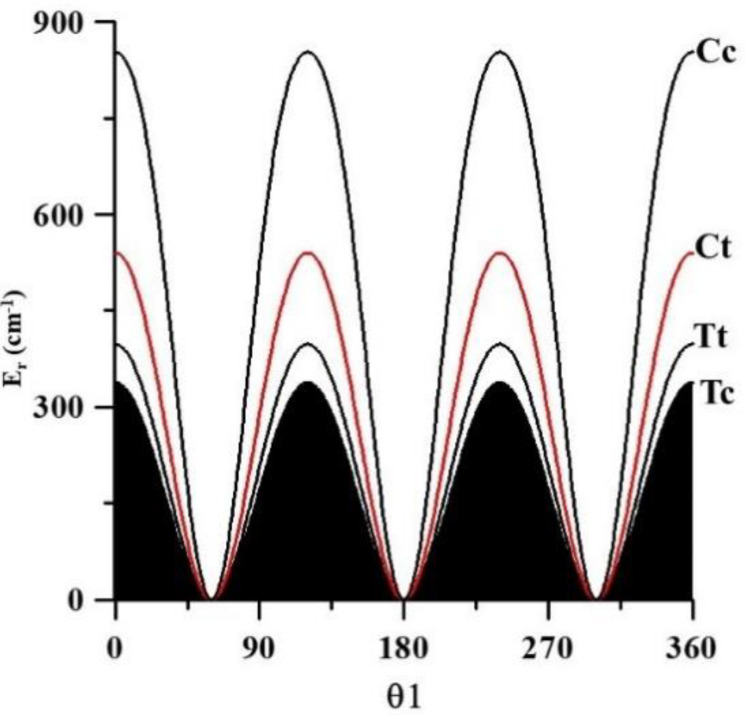
CH_3_ torsional barrier.

The methyl torsional barrier of Cc computed using CCSD(T)-F12, is strongly affected by the repulsive interactions. We calculated it to be 852.8 cm^−1^, whereas the corresponding values for Tc, Tt, and Ct are 338.0, 397.8, and 540.2 cm^−1^, respectively (see Figure 3). The computed barrier corresponding to Tc is a very good agreement with the experimental value of 336.358(50) cm^−1^, obtained by Kisiel et al. [12] in the analysis of the A and E lines of the millimeter spectrum.

**Figure 4:**
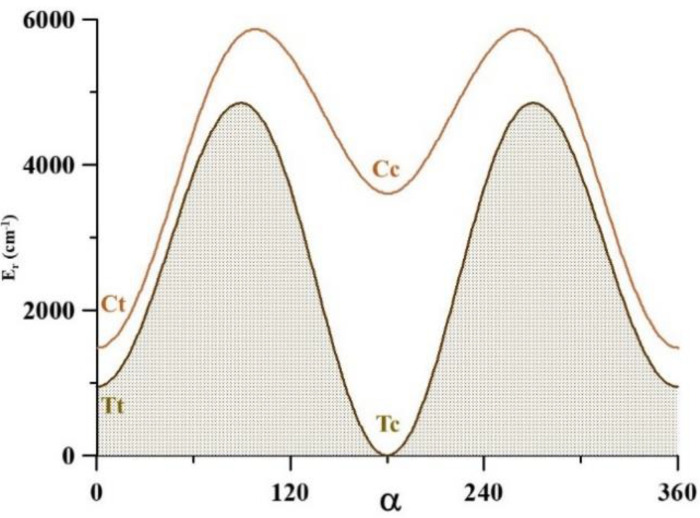
The CCSD(T)-F12 V^OH^ torsional barriers. The Tc **→** Tt and Ct **→** Cc pathways were computed using the θ_1_ and θ_2_ values of the Tc and Ct conformers, respectively.

**Figure 5:**
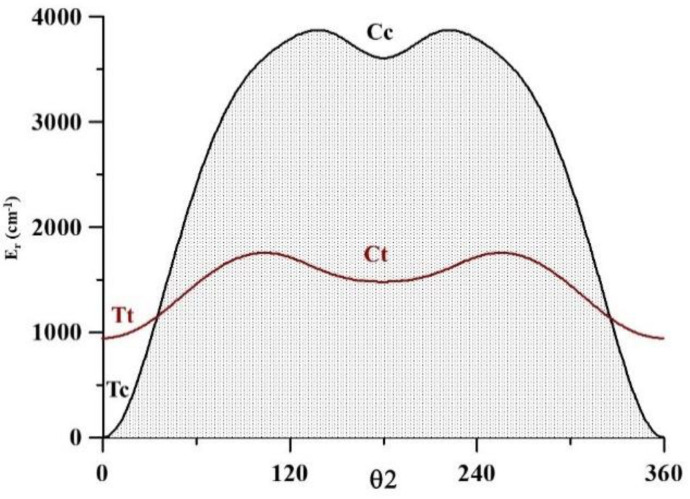
The CCSD(T)-F12 V^CC^ torsional barriers. The Tc **→** Cc and Tt → Ct pathways were computed using the θ_1_ and α values of the Tc and Tt conformers, respectively.

The torsional barriers V^OH^ and V^CC^, shown in Table 1 and Figure 4 and Figure 5, restrict the t → c and T → C interconversions. They were computed from the vibrationally corrected three-dimensional potential energy surface described below. The one-dimensional cuts were obtained by fixing two coordinates at their values in the low-energy conformer involved in the pathways. With the exception of V^CC^ located between Tt and Ct, all the barriers are higher than 3000 cm^−1^. The pathways emphasize the instability of the Cc that easily transforms in Ct or Tc.

Whereas the Tt → Ct process can occur at very low temperatures, the Tc → Tt pathway is impeded by a relatively high barrier. It may be inferred that the very low vibrational energy levels of the most stable conformer, Tc, can be studied considering it as a molecule with a unique minimum. However, since the conformers Tt and Ct are very close in energy (ΔE = 538 cm^−1^) and the barriers restricting the processes Tt → Ct and Ct → Tt are relatively low (812 and 274 cm^−1^), an accurate calculation of the low vibrational energy levels requires taking into consideration the tunneling effects in those barriers.

In Table 3, the Tc anharmonic wavenumbers are compared with the observed bands assigned by Hollenstein et al. [17], who measured the IR spectrum in the 4000–200 cm^−1^ region, and with the mid-IR vapor-phase spectrum of Plath et al. [8]. Emphasized in bold are the transitions in which important Fermi displacements are predicted by the VPT2 theory. The OH stretching fundamental denotes the presence of the intramolecular hydrogen bond in the Tc conformer. The frequency, 3426 cm^−1^, observed at 3463 cm^−1^ [8], is lower than the one of Tt (calculated to be 3561 cm^−1^ and observed at 3579 cm^−1^ [8]) and the one of Ct (3554 cm^−1^). In Cc, the frequency is higher than in the remaining structures (3636 cm^−1^) due to the proximity of the methyl group hydrogens. Two internal coordinates, the H10O9C3O8 dihedral angle and the out-of-plane C=O bending contribute to the ν_21_ mode, computed to be 606 cm^−1^ and observed by Hollenstein et al. [17] at 668 cm^−1^. In this paper, the H10O9C3O8 dihedral angle is identified with the OH torsional coordinate α. Direct measurements of the CH_3_ torsional fundamental are not available.

### 2.3. Ground Vibrational State: Rotational and Centrifugal Distortion Constants

The ground vibrational state rotational constants and the centrifugal distortion constants of the four conformers are shown in Table 4. The collected distortion constants are parameters of the asymmetrically reduced Hamiltonian in the I ^r^ representation [37]. They were determined using two different basis sets, AVTZ and the corresponding triple zeta basis set ignoring the diffuse functions, VTZ. For the low-lying conformer, Tc, the MP2/VTZ parameters were obtained in a very good agreement with those fitted by Kisiel et al. [12] during the assignment of the millimeter-wave spectrum using the ERHAM program [38]. Their analysis included more than 1500 lines comprising A and E internal sublevels. Given the good agreement between ab initio and experimental results for Tc, it can be expected that the MP2/VTZ predicted sets will be useful for future assignments of the remaining conformers.

The ground vibrational state rotational constants were computed using the CCSD(T)-F12 equilibrium parameters of Table 2 and the following equation, proposed and verified in previous studies [22,39,40,41].

B_i0_ = B_ie_ (CCSD(T)-F12/AVTZ-F12) + ∆B_i_^core^ (CCSD(T)/CVTZ) + ∆Bi_vib_ (MP2/AVTZ); i = a;.b;.c (2)

Here, ∆B_ie_^core^ takes into account the core-valence-electron correlation effect on the equilibrium parameters. It can be evaluated as the difference between B_ie_(CV) (calculated correlating both core and valence electrons) and B_ie_(V) (calculated correlating just the valence electrons). For Tc, the correction increases 15.8, 11.8, and 6.9 MHz the equilibrium values of A, B, and C, respectively. ∆B_ivib_ represents the vibrational contribution to the rotational constants derived from the VPT2 α_ir_ vibration-rotation interaction parameters.

The rotational constants of the preferred conformer Tc are in a very good agreement with the parameters fitted by Kisiel et al. [12] (A_0_^CAL^ − A_0_^EXP^ = −4.96 MHz, B_0_^CAL^ − B_0_^EXP^ = −2.23 MHz, and C_0_^CAL^ − C_0_^EXP^ = −1.78 MHz). Generally, for many molecules, if Equation (2) is applied, the computed B_0_ and C_0_ are more accurate than A_0_ [41]. Based on the very good agreement between our calculated rotational constants for Tc conformer and their experimentally determined counterparts, such parameters for the other isomers are also plausibly very accurate.

### 2.4. The Far Infrared Region

The ground electronic state potential energy surface of pyruvic acid presents a total of 12 minima corresponding to four conformers since each methyl group inter-transforms into three equivalent minima. Then, our exploration of the far infrared region uses a three-dimensional variational procedure that takes into account the interconversion of the minima. The three torsional coordinates of Equation (1) that are responsible for the non-rigidity are defined to be the independent variables. The validity of the separability of the three internal rotations θ_1_, θ_2_ and α from the remaining vibrations entails a previous discussion. Although VPT2 does not represent the right theory for internal rotation studies, it provides a valuable preliminary description since the three internal rotation coordinates represent the most relevant contributions to three normal modes. The anharmonic analysis of Table 3 can supply arguments based on energies and the cubic force field. In the most stable conformer, pyruvic acid displays five out-of-plane fundamental transitions lying below 1000 cm^−1^. The central bond torsion and the methyl group torsion lie at very low frequencies and interact. Both motions contribute to two normal modes, ν_23 and_ ν_24_ (ν_24_^(VPT2)^ = 93 cm^−1^; ν_23_^(VPT2)^ = 124 cm^−1^). In principle, it can be assumed that at least, three excited CH_3_ and four excited C-C torsional states could be computed using a two-dimensional procedure since the VPT2 resulting energies are lower than the next lowest frequency mode. However, above 350 cm^−1^, three out-of-plane fundamentals, ν_22_^(VPT2)^ = 392 cm^−1^, ν_21_^(VPT2)^ = 606 cm^−1^, and ν_20_^(VPT2)^ = 792 cm^−1^, have been found. Although these modes cannot really be interpreted in terms of local modes, they can be interpreted as two skeletal bending modes and to the OH torsional fundamental. It cannot be strictly stated that ν_21_ represents the OH torsional fundamental, since the out-of-plane bending of the neighboring C=O group has an important contribution to ν_21_ [17]. In addition, the OH torsional mode is strongly coupled to the C-C torsional mode, ν_24_. In the most stable Tc conformer, the OH hydrogen forms part of an intramolecular hydrogen bond that vanishes with the C-C torsional mode excitations. To obtain accurate low energy levels for the two low-lying modes, ν_24_ and ν_23_, the internal coordinate describing the OH torsion must be considered explicitly as an independent variable.

If the 3D model is applied, very accurate CH_3_ and C-C torsional levels and very unrealistic OH torsional levels will be expected. In addition, the VPT2 theory predicts a displacement of the 2ν_23_ overtone (∆(2ν_23_) ~ −5 cm^−1^) due to Fermi resonances with the ν_16_ fundamental which is not defined as an independent variable. The remaining low-lying levels seem to be free of relevant resonances.

The 3D Hamiltonian for J = 0 must be defined as [34,35,36]:

(3) $$\begin{array}{c} H\left(\theta_{1},\theta_{2},\alpha \right)=-\overset{3}{\underset{i=1}{\sum }}\overset{3}{\underset{j=1}{\sum }}\left(\frac{\partial }{\partial q_{i}}\right)B_{q_{i}q_{j}}\left(\theta_{1},\theta_{2},\alpha \right)\left(\frac{\partial }{\partial q_{j}}\right)\;+V^{eff}\left(\theta_{1},\theta_{2},\alpha \right)\\ q_{i}=\theta_{1},\;\theta_{2},\alpha \;;\;q_{j}=\theta_{1},\;\theta_{2},\alpha \end{array}$$

where B_qiqj_ (θ_1_, θ_2_, α) are the kinetic energy parameters [35,36]; the effective potential is the sum of three terms:

V^eff^ (θ_1_, θ_2_, α) = V(θ_1_, θ_2_, α) + V’(θ_1_, θ_2_, α) + V^ZPVE^(θ_1_, θ_2_, α) (4)

V(θ_1_, θ_2_, α), V’(θ_1_, θ_2_, α), and V^ZPVE^(θ_1_, θ_2_, α) represent the ab initio potential energy surface, the Podolsky pseudopotential, and the zero point vibrational energy correction, respectively.A grid of 332 geometries was selected to determine the Hamiltonian parameters. In all these geometries, the remaining 3Na-9 internal coordinates (Na = number of atoms) were optimized at the MP2/AVTZ level of theory, whereas three internal coordinates were fixed at the following values:

H5C2C1C3 = 0, 90, 180, and −90° O8C2C1C3 = 0, 30, 60, …, 150, 180° H10O9C3O8 = −180, −150,…, 0…, 150, 180°(5)

Single point CCSD(T)-F12/AVTZ-F12 calculations were performed on the 332 optimized geometries. The total electronic energies were fitted to a symmetry adapted triple Fourier series to obtain the V(θ_1_, θ_2_, α) ab initio potential energy surface (R^2^ = 0.99999; σ = 0.013 cm^−1^). The following analytical expression was employed:

(6) $$\begin{array}{c} V^{\;}\left(\theta_{1},\theta_{2},\;\alpha \right)=\underset{m=0}{\sum }\underset{n=0}{\sum }\underset{l=0}{\sum }\;\;\;(A_{nml}^{ccc}\cos3m\cos{n\theta }_{2}\cos l\alpha \;\\ +A_{nml}^{ssc}\sin3{m\theta }_{1}\sin{n\theta }_{2}\cos l\alpha \\ +A_{nml}^{css}\cos3{m\theta }_{1}\sin{n\theta }_{2}\sin l\alpha \\ +A_{nml}^{scs}\sin3{m\theta }_{1}\cos{n\theta }_{2}\sin l\alpha ) \end{array}$$

**Figure 6:**
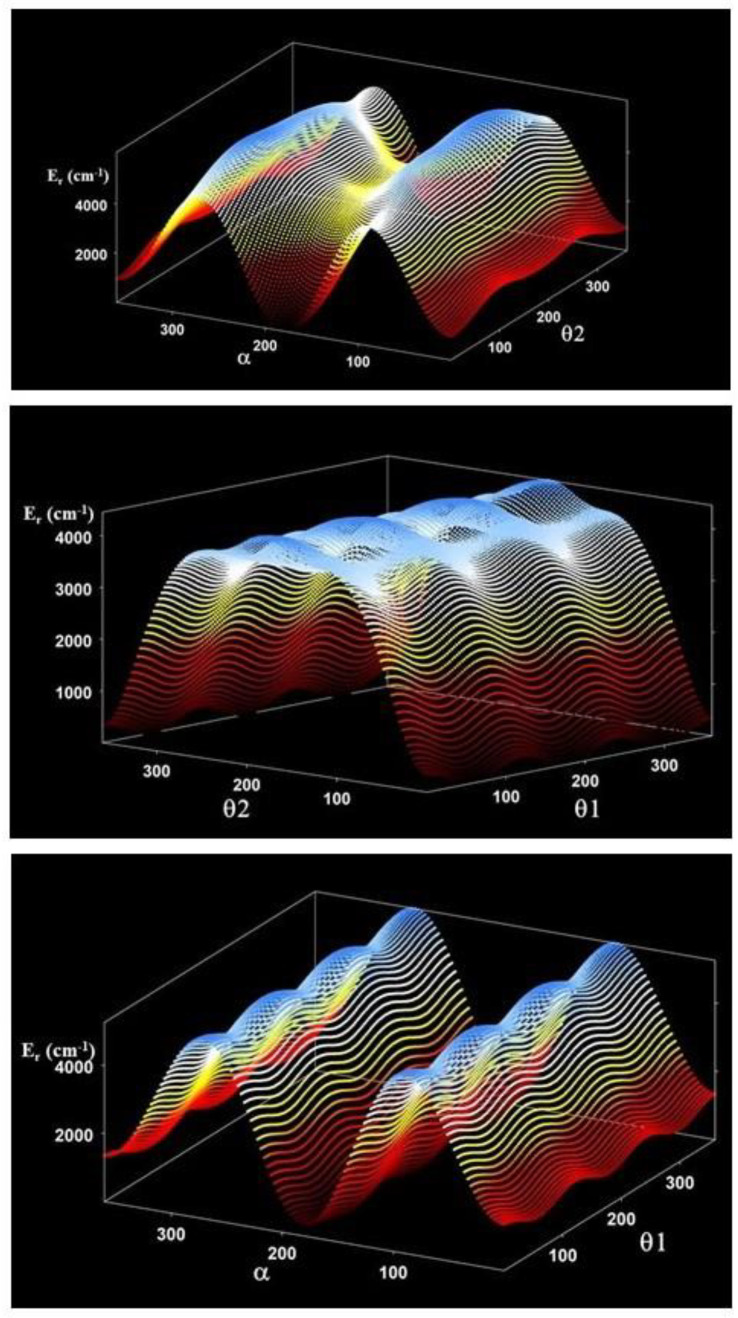
Two-dimensional cuts of the 3D potential energy surface of pyruvic acid.

Formally identical expressions were employed for V’(θ_1_, θ_2_, α) and V^ZPVE^(θ_1_, θ_2_, α). The pseudopotential represents a negligible correction. However, the zero point vibrational correction V^ZPVE^(θ_1_, θ_2_, α) is mandatory. It was computed from the MP2/AVTZ harmonic fundamentals using all the geometries. The expansion coefficients of the final effective potential are supplied as supplementary material in Table S1. Two dimensional cuts of the potential energy surface are represented in Figure 6.

A formally identical formula was also used for the kinetic energy parameters which were computed for the 332 partially optimized geometries. Details on the procedure for obtaining the Hamiltonian parameters using ENEDIM [34,35,36]. The $A_{000}^{ccc}$ coefficients of the kinetic parameters are given in Table 5.

The symmetry adapted triple Fourier series were employed as trial functions. To reduce dimensionality, a contracted basis set was employed for the C-C torsional coordinate performing a pre-diagonalization of the Hamiltonian matrix [27,42]. This route helps the classification of the resulting energies.

The minimum number of functions required for convergence produces a matrix of 169,455 × 169,455 elements. The symmetry factorizes it into four blocks with the dimensions as 28,243 (A_1_), 28,242 (A_2_), and 56,485 × 2 (E). Using the contracted basis set, the dimensions are reduced to 4648 (A_1_), 4647 (A_2_), and 9295 × 2 (E). This represents the 16% of the original matrix.

Each energy level splits into three subcomponents corresponding to a non-degenerate representation (A_1_ and A_2_) and to the double-degenerate representation, E. Table 6 collects torsional energies up to ~425 cm^−1^ over the ground vibrational state (ZPVE = 406.072 cm^−1^). They are compared with previous data from Ref. [16] and with computations performed in this work using VPT2. All the collected levels were assigned to the Tc conformers since the first secondary minimum lies at 877.5 cm^−1^. The energy levels of Ref. [16] were obtained from experimental splittings using a flexible two-dimensional model.

More than 50 torsional energy levels (including subcomponents) were localized in the 406–986 cm^−1^ region below the first excited state (0 0 1) of the OH torsion ((0 0 1) = 581.306 cm^−1^ (A_2_) and 581.327 cm^−1^ (E)). All of the first 50 energies represent excitations of the ν_24_ and ν_23_ modes. Since the classification of the resulting levels is really difficult given the large density of states in the same region, procedures developed for previous studies were employed for a right assignment. The properties of the 3D-wavefunctions and the contribution of the contracted basis functions [27,42] allow assigning the main part of the energies. The assignments of few E components (in blue in Table 6) are not conclusive.

The A_1_/E splitting of the ground vibrational state has been evaluated to be 0.024 cm^−1^ as it was expected given the high of the methyl torsional barrier (338.0 cm^−1^). This value is in a very good agreement with the results of Refs. [12,16] (0.7203 GHz [16], 0.72355(43) GHz) [12]. It can be emphasized that a surprising very good agreement between our calculations and the results of these previous references, was found for many vibrational and rotational properties [12,16].

The ν_24_ fundamental (010 **←** 000) computed to be 93 cm^−1^ with VPT2, presents two components at 89.754 cm^−1^ (A_2_ **←** A_1_) and 89.709 cm^−1^ (E **←** E) in a very good agreement with Refs. [16,17]. The methyl torsional fundamental ν_23_ (100 **←** 000) computed to be 123 cm^−1^ with VPT2, presents two components at 120.539 cm^−1^ (A_2_ **←** A_1_) and 119.843 cm^−1^ (E **←** E). Previous estimations placed it in the 118.2–117.7 cm^−1^ zone.

With respect to the results of Ref. [16], there is a disagreement for the relative order of the levels (1 1 0) and (2 0 0). The VPT2 theory predicts Fermi displacements of the 2ν_23_ overtone.

## 3. Discussion

In general, the computed spectroscopic properties corresponding to the most stable conformer of pyruvic acid (rotational and torsional parameters) are in a surprising good agreement with previous experimental data. This behavior was not expected since molecules with low methyl torsional barriers can present sudden problems causing divergences between “ab initio” results and observations. On the basis of this agreement, we predict parameters for all the conformers to be employed in other spectrum assignments.

Four equilibrium geometries of pyruvic acid outcome from the search with CCSD(T)-F12. The preferred geometry Tc stabilizes by the formation of an intramolecular hydrogen bond (H10….O4 = 2.0030 Å) that a play a role in the internal dynamics of the molecule. Two next conformers, Tt and Ct, lie between 950 and 2000 cm^−1^ region over Tc. The higher energy conformer Cc is very unlikely to be observed. With few exceptions, all the V^OH^ and V^CC^ barriers restricting the conformers interconversions are higher than 3000 cm^−1^. The interconversion pathways emphasize the instability of the Cc that easily transforms to Ct or Tc.

More than 50 torsional energy levels (including subcomponents) were localized in the 406–986 cm^−1^ region below the first excited state (0 0 1) of the OH torsional mode. They represent excitations of the ν_24_ and ν_23_ modes and all of them are assigned to the most stable conformer. The energies can be calculated with accuracy using a three-dimensional model although the VPT2 theory predicts Fermi displacements of the 2ν_23_ overtone caused by resonances that are not considered.

In general, there is an excellent agreement for the fundamental transitions and for the splittings between the new calculations and the previous experimental or semiempirical data. However, there is not an agreement for the relative order of the levels (1 1 0) and (2 0 0). We propose a new assignment for these two energies to be taken into consideration in future spectrum assignments.

## 4. Materials and Methods

The Materials and Methods are detailed in the section dedicated to the “Electronic structure calculations”.

## Figures and Tables

**Table 1:** CCSD(T)-F12/AVTZ-F12 relative energies (E, E^ZPVE^, in cm^−1^), internal rotation barriers (V_3_, V^OH^, V^CC^ in cm^−1^), and equilibrium rotational constants (in MHz); MP2/AVTZ dipole moment (in D) of pyruvic acid.

		Tc	Tt	Ct	Cc
	Calc.	Exp. [Ref]	Calc.	Calc.	Calc.
E	0.0 ^a^		960.3	1526.0	3742.6
E^ZPVE^	0.0 ^b^		877.5	1416.3	3563.5
θ_1_	180°		180°	180°	180°
θ_2_	0°		0°	180°	180°
α	180°		0°	0°	180°
					
A_e_	5537.641		5640.946	5568.843	5513.683
B_e_	3606.390		3497.767	3503.128	3483.147
C_e_	2213.503		2187.875	2179.097	2163.620
					
μ_a_	2.5575	2.27 ± 0.02 [14]	0.3809	0.2021	2.9383
μ_b_	0.131	0.35 ± 0.02 [14]	1.353	4.4313	5.2582
μ_c_	0.0		0.0	0.0	0.0
μ	2.5609	2.3 ± 0.03 [14]	1.4056	4.4359	6.0235
					
V_3_	338.0	336.358(50) [12]	397.8	540.2	852.8
V^OH^ (Tc → Tt)			4852		
V^OH^ (Ct → Cc)			4383		
V^CC^ (Tc → Cc)			3871		
V^CC^ (Tt → Ct)			812		

E = −342.03025 a.u.; ZPVE = 15601.9 cm^−1^.

**Table 2:** CCSD(T)-F12/AVTZ-F12 equilibrium structural parameters (distances, in Å, angles, in degrees) of pyruvic acid conformers.

	Tc	Tt	Ct	Cc
H10….O4	2.0030	-	-	-
C1C2	1.4971	1.5008	1.5031	1.5149
C1C3	1.5433	1.5408	1.5476	1.5561
O4C1	1.2168	1.2063	1.2048	1.2031
H5C2	1.0870	1.0863	1.0866	1.0865
H6C2	1.0914	1.0908	1.0909	1.0935
H7C2	1.0914	1.0908	1.0909	1.0935
O8C3	1.2069	1.2054	1.1972	1.1922
O9C3	1.3363	1.3353	1.3502	1.3536
H10O9	0.9734	0.9670	0.9671	0.9616
C2C1C3	116.8	114.7	117.8	118.8
O4C1C3	117.8	120.3	117.8	118.2
H5C2C1	109.9	109.2	109.0	108.9
H6C2C1	109.3	109.7	109.9	110.6
H7C2C1	109.3	109.7	109.9	110.6
O8C3C1	122.9	122.6	124.2	122.3
O9C3O8	124.6	124.8	124.4	121.6
H10O9C3	105.3	106.5	106.6	110.8
H6C2C1H5	−121.9	−121.5	−121.3	−119.9
H7C2C1H5	121.9	121,5	121.3	119.9

**Table 3:** MP2/AVTZ anharmonic fundamentals ^a^ (in cm^−1^).

		Tc		Tt		Ct	Cc
		Calc.	Exp. ^ref^	Calc.	Exp. ^ref^	Calc.	Calc.
ν(a’)							
ν_1_	OH st	3426	3463 ^b^	3561	3579 ^b^	3554	3636
ν_2_	CH_3_ st	3072	3025 ^b^	3072		3067	3059
ν_3_	CH_3_ st	2971	2941 ^b^	2973		2973	2958
ν_4_	C3=0 st	1782	1804 ^b^	1747		1773	1788
ν_5_	C1=0 st	1705	1737 ^b^	1723		1721	1721
ν_6_	CH_3_ b	1435	1424 ^b^	1436		1436	1447
ν_7_	C-C- st	1380	1391 ^b^	1382		1363	1355
ν_8_	CH_3_ b	1332	1360 ^b^	1356		1340	1273
ν_9_	COH b	1214	1211 ^b^	1203		1162	1177
ν_10_	C-OH st	1135	1133 ^b^	1120		1102	1098
ν_11_	CH_3_ b	971	~970 ^c^	962		971	965
ν_12_	C-C st	756	761 ^c^	731		724	738
ν_13_	skeletal b	602	604 ^c^	587		605	606
ν_14_	skeletal b	529		517		484	478
ν_15_	OCCb	387		386		401	405
ν_16_	CCCb	258		245		253	263
ν(a”)							
ν_17_	CH_3_ st	3019		3024		3026	3005
ν_18_	CH_3_ b	1432		1437		1441	1458
ν_19_	CH_3_ b	1017	1030 ^b^	1019		1020	1011
ν_20_	skeletal b	792		723		713	699
ν_21_	OH tor (+C=O)	606	668 ^c^	601		601	461
ν_22_	skeletal b	391	392 ^c^	373		373	357
ν_23_	CH_3_ tor	123		130		151	187
ν_24_	C-C tor	93	90 ^c^	39		19	1

^a^ Emphasized in bold transitions in which important Fermi displacements are predicted. ^b^ Ref. [8]: Infrared absorption spectroscopy. ^c^ Ref. [17]: Infrared spectroscopy in an argon matrix.

**Table 4:** Ground vibrational state rotational constants (in MHz) and centrifugal distortion constants corresponding to the asymmetrically reduced Hamiltonian parameters (I^r^ representation) ^a^.

		Tc	Tt	Ct	Cc
in MHz					
	Calc.	Exp. [12]	Calc.	Calc.	Calc.
A_0_	5530.506	5535.46113 (18)	5629.841	5553.752	5499.149
B_0_	3581.177	3583.408634 (78)	3473.363	3474.982	3449.328
C_0_	2203.077	2204.858443 (63)	2180.423	2173.717	2158.751
in kHz					
Δ_J_	0.645465	0.675114 (35)	0.577981	0.583771	1.831225
Δ_K_	1.259501	1.49373 (49)	1.275684	1.340695	3.880986
Δ_JK_	−0.550295	−0.77911 (12)	−0.392555	−0.469353	−4.231851
δ_J_	0.252245	0.264241 (16)	0.221177	0.225223	0.480955
δ_K_	0.582614	0.552491 (97)	0.601105	0.576154	5.552698
in Hz					
H_J_	0.000111	0.0001239 (86)	0.000116	0.000118	−0.320165
H_K_	0.003486	0.00561 (48)	0.004440	0.010283	−6.821416
H_JK_	0.000466	-	0.000523	0.001860	−2.315587
H_KJ_	−0.003375	−0.00171 (13)	−0.004398	−0.011623	9.438257
φ_J_	0.000091	0.0000718 (46)	0.000094	0.000098	−0.252688
φ_JK_	0.002459		0.003983	0.011783	−7.667262
φ_K_	0.000755	0.002441 (76)	0.000772	0.001065	−0.425569

^a^ The rotational constants were computed using Equation (2) and the centrifugal distortion constants using a MP2/VTZ force field.

**Table 5:** $A_{000}^{ccc}$ coefficients of the kinetic energy parameters (in cm^−1^).

	$A_{000}^{ccc}$		$A_{000}^{ccc}$
B_θ1θ1_	5.4904	B_θ1θ2_	−0.1751
B_θ2θ2_	0.7411	B_θ1α_	0.0376
B_αα_	18.5985	B_θ2α_	0.0061

**Table 6:** Low-lying torsional energy levels (in cm^−1^) of pyruvic acid computed variationally and/or with VPT2 ^a^. The m, n, and l quanta corresponds to the θ_1,_ θ_2_, and α coordinates.

m n l	Variational		VPT2	Ref. [16]	m n l	Variational		VPT2
0 0 0	A_1_ E	0.000 ^b^ 0.024	-	0.000 0.024	3 0 0 (3ν_23_)	A_2_ E	327.476 278.686	340
								
0 1 0 (ν24)	A2 E	89.754 89.709	93	90.1 90.0	4 0 0 (4ν23)	A_1_ E	334.735 394.098	432
1 0 0 (ν_23_)	A_2_ E	120.539 119.833	123	118.3 117.7	(ν_24_ν_16_)			348
0 2 0 (2ν_24_)	A_1_ E	178.744 178.766	186	179.9 180.0	(ν_23_ν_16_)			380
1 1 0 (ν_24_ν_23)_	A_1_ E	202.976 206.570	214	222.5 225.0	0 4 0 (4ν_24_)	A_1_ E	355.311 355.197	368
2 0 0 (2ν_23_)	A_1_ E	223.301 226.756	232	200.5 204.5	(ν_15_)			387
(ν_16_)			258		(ν_22_)			391
0 3 0 (3ν_24_)	A_2_ E	267.193 267.161	277		1 3 0 (3ν_24_ν_23_)	A_1_ E	380.268 390.184	393
1 2 0 (2ν_24_ν_23_)	A_2_ E	291.127 298.695	304		2 2 0 (2ν_23_2ν_24_)	A_1_ E	408.508 421.158	412
2 1 0 (2ν_23_ν_24_)	A_2_ E	315.337 325.067	325		3 1 0 (3ν_23_ν_24_)	A_1_ E	425.414 368.775	425
					0 0 1 (ν_21_)	A_2_ E	581.306 581.327	680

^a^ Emphasized in bold transitions in which important Fermi displacements are predicted (2ν_23_ ↔ ν_16_, ν_24_ν_22_ ↔ ν_14_). In italic and blue are energies in which the assignments are not conclusive (ZPVE = 406.072 cm^−1^).

## Data Availability

Not applicable.

## Supplementary Materials

The following are available online at Table S1: Expansion coefficients of the potential energy surface (in cm^−1^).

## Sample Availability

The parameters of the torsional Hamiltonian are available from the authors.

## References

[1] Yu S. (2000). Role of organic acids (formic, acetic, pyruvic and oxalic) in the formation of cloud condensation nuclei (CCN): A review. Atmos. Res..

[2] Mellouki A., Wallington T.J., Chen J. (2015). Atmospheric chemistry of oxygenated volatile organic compounds: Impacts on air quality and climate. Chem. Rev..

[3] Nozière B., Kalberer M., Claeys M. (2015). The molecular identification of organic compounds in the atmosphere: State of the art and challenges. Chem. Rev..

[4] Reed Harris A.E., Pajunoja A., Cazaunau M., Gratien A., Pangui E., AMonod A., Griffith E.C., Virtanen A., Doussin J.-F., Vaida V. (2017). Multiphase photochemistry of pyruvic acid under atmospheric conditions. J. Phys. Chem. A.

[5] Church J.R., Vaida V., Skodje R.T. (2020). Gas-phase reaction kinetics of pyruvic acid with OH radicals: The role of tunneling, complex formation, and conformational structure. J. Phys. Chem. A.

[6] Church J.R., Vaida V., Skodje R.T. (2021). Kinetic study of gas-phase reactions of pyruvic acid with HO_2_. J. Phys. Chem. A.

[7] Takahashi K., Plath K.L., Skodje R.T., Vaida V. (2008). Dynamics of vibrational overtone excited pyruvic acid in the gas phase: Line broadening through hydrogen-atom chattering. J. Phys. Chem. A.

[8] Plath K.L., Takahashi K., Skodje R.T., Vaida V. (2009). Fundamental and overtone vibrational spectra of gas-phase pyruvic acid. J. Phys. Chem. A.

[9] Sephton M.A. (2002). Organic compounds in carboniceous meteorites. Nat. Prod. Rep..

[10] Mehringer D.M., Snyder L.E., Miao Y., Lovas F. (1997). Detection and confirmation of interstellar acetic acid. Astrophys. J..

[11] Kleimeier N.F., Eckhardr A.K., Schreiner P.R., Kaiser R.I. (2020). Interstellar formation of biorelevant pyruvic acid (CH_3_COCOOH). Chem.

[12] Kisiel Z., Pszczołkowski L., Białkowska-Jaworska E., Charnley S.B. (2007). The millimeter wave rotational spectrum of pyruvic acid. J. Mol. Spectrosc..

[13] Kaluza C.E., Bauder A., Günthard H.H. (1973). The microwave spectrum of pyruvic acid. Chem. Phys. Lett..

[14] Marstokk K.-M., Möllendal H. (1974). Microwave spectrum, conformation, barrier to internal rotation and dipole moment of pyruvic acid. J. Mol. Struct..

[15] Dyllick-Brenzinger C.E., Bauder A., Günthard H.s.H. (1977). The substitution structure, barrier to internal rotation, and low frequency vibrations of pyruvic acid. Chem. Phys..

[16] Meyer R., Bauder A. (1982). Torsional coupling in pyruvic acid. J. Mol. Spectrosc..

[17] Hollenstein H., Akermann F., Gunthard H.H. (1978). Vibrational analysis of pyruvic acid and D-, ^13^C- and ^18^O-labelled species: Matrix spectra, assignments, valence force field and normal coordinate analysis. Spectrochim. Acta A.

[18] Reva I., Nunes C.M., Biczysko M., Fausto R. (2015). Conformational switching in pyruvic acid isolated in Ar and N_2_ matrixes: Spectroscopic analysis, anharmonic simulation, and tunneling. J. Phys. Chem. A.

[19] Tarakeshwar P., Manogaran S. (1998). An ab initio study of pyruvic acid. J. Mol. Struct..

[20] Barone V., Biczysko M., Bloino J. (2015). CC/DFT Route toward accurate structures and spectroscopic features for observed and elusive conformers of flexible molecules: Pyruvic acid as a case study. J. Chem. Theory Comput..

[21] Senent M.L. (2001). Ab initio determination of the torsional spectra of acetic acid. Mol. Phys..

[22] Dalbouha S., Mogren Al-Mogren M., MLSenent M.L. (2021). Rotational and torsional properties of various monosubstituted isotopologues of acetone (CH_3_-CO-CH_3_) from explicitly correlated ab initio methods. ACS Earth Space Chem..

[23] Senent M.L., Moule D.C., Smeyers Y.G., Toro-Labbé T., Peñalver F.J. (1994). A theoretical spectroscopic study of the Ã^1^A_u_(S_1_) ← X^1^A_g_(S_0_), n → π* Transition in Biacetyl, (CH_3_CO)_2_. J. Mol. Spectrosc..

[24] Knizia G., Adler T.B., Werner H.-J. (2009). Simplified CCSD(T)-F12 methods: Theory and benchmarks. J. Chem. Phys..

[25] Werner H.-J., Adler T.B., Manby F.R. (2007). General orbital invariant MP2-F12 theory. J. Chem. Phys..

[26] Senent M.L., Ruiz R., Dominguez-Gómez R., Villa M. (2009). CCSD(T) study of the far-infrared spectrum of ethyl methyl ether. J. Chem. Phys..

[27] Boussesi R., Senent M.L. (2020). Computational analysis of the far Infrared spectral region of various deuterated varieties of Ethylene Glycol. Phys. Chem. Chem. Phys..

[28] Werner H.-J., Knowles P.J., Manby F.R., Schütz M., Celani P., Knizia G., Korona T., Lindh R., Mitrushenkov A., Rauhut G. (2012). MOLPRO.

[29] Kendall R.A., Dunning T.H., Harrison R.J. (1992). Electron affinities of the first-row atoms revisited. Systematic basis sets and wave functions. J. Chem. Phys..

[30] Knowles P.J., Hampel C., Werner H.-J. (1993). Coupled cluster theory for high spin, open shell reference wave functions. J. Chem. Phys..

[31] Woon D.E., Dunning T.H. (1995). Gaussian basis sets for use in correlated molecular calculations. V. Core-valence basis sets for boron through neon. J. Chem. Phys..

[32] Barone V. (2005). Anharmonic vibrational properties by a fully automated second-order perturbative approach. J. Chem. Phys..

[33] Frisch M.J., Trucks G.W., Schlegel, Scuseria G.E., Robb M.A., Cheeseman J.R., Scalmani G., Barone V., Petersson G.A., Nakatsuji H. (2016). Gaussian 16, Revision, C.01.

[34] Senent M.L. (2001). ENEDIM, “A Variational Code for Non-Rigid Molecules”. http://tct1.iem.csic.es/PROGRAMAS.htm.

[35] Senent M.L. (1998). Determination of the kinetic energy parameters of non-rigid molecules. Chem. Phys. Lett..

[36] Senent M.L. (1998). Ab initio determination of the roto-torsional energy levels of trans-1,3-butadiene. J. Mol. Spectrosc..

[37] Watson J.K.G. (1968). Determination of Centrifugal Distortion Coefficients of Asymmetric-Top Molecules. III. Sextic Coefficients. J. Chem. Phys..

[38] Groner P. (1997). Effective rotational Hamiltonian for molecules with two periodic large-amplitude motions. J. Chem. Phys..

[39] Boussesi R., Senent M.L., Jaïdane N. (2016). Weak intramolecular interaction effects on the low temperature spectra of ethylene glycol, an astrophysical species. J. Chem. Phys..

[40] Motiyenko R.A., Margulès L., Senent M.L., Guillemin J.C. (2018). Internal rotation of OH group in 4-hydroxy-2-butynenitrile studied by millimeter-wave spectroscopy. J. Phys. Chem. A.

[41] Dalbouha S., Senent M.L., Komiha N., Domínguez-Gómez R. (2016). Structural and spectroscopic characterization of methyl isocyanate methyl cyanate, methyl fulminate, and acetonitrile N-oxide using highly correlated ab initio methods. R. J. Chem. Phys..

[42] Gámez V., Senent M.L. (2021). The formation of C_3_O_3_H_6_ structural isomers in the gas phase through barrierless pathways. Formation and spectroscopic characterization of methoxy acetic acid. Astrophys. J..

