# Supplementary material for: Large Amplitude Motions of Pyruvic Acid (CH3-CO-COOH)
Source: Molecules. 2021 Jul 14;26(14):4269. doi: 10.3390/molecules26144269 (PMC8303138; doi:10.3390/molecules26144269)
Supplement: Supplementary file 1 [file molecules-26-04269-s001.zip › molecules-1300628-supplementary.pdf]

TABLE S1: Expansion coefficients of the potential energy surface (in cm^{-1})

M=3m $M \geq 0 \Rightarrow \cos M\theta_1$; $M < 0 \Rightarrow \sin M\theta_1$;
$n \geq 0 \Rightarrow \cos n\theta_2$; $n < 0 \Rightarrow \sin n\theta_2$;
$l \geq 0 \Rightarrow \cos l\alpha$; $l < 0 \Rightarrow \sin l\alpha$;

	M	n	l		M	n	l
3969.948	0	0	0	204.105	3	0	0
-2.325	6	0	0	-740.204	0	1	0
-302.522	0	2	0	-78.951	0	3	0
21.877	0	4	0	0.794	0	5	0
0.275	0	6	0	-568.232	0	0	1
-1710.005	0	0	2	-37.487	0	0	3
19.622	0	0	4	-1.837	0	0	5
-0.121	0	0	6	-18.854	3	1	0
23.476	3	2	0	-38.249	3	3	0
23.756	3	4	0	-0.852	3	5	0
1.100	3	6	0	2.209	6	1	0
-3.456	6	2	0	3.037	6	3	0
-3.046	6	4	0	1.321	6	5	0
-1.702	6	6	0	43.574	-3	-1	0
-27.854	-3	-2	0	43.951	-3	-3	0
-23.766	-3	-4	0	-0.406	-3	-5	0
6.159	3	0	1	1.512	3	0	2
-0.041	3	0	3	0.122	3	0	4
-0.926	3	0	5	0.192	3	0	6
-0.225	6	0	1	-0.360	6	0	2
0.054	6	0	3	-0.293	6	0	4
0.360	6	0	5	-0.122	6	0	6
-0.009	-3	0	-1	0.472	-3	0	-2
0.001	-3	0	-3	-0.204	-3	0	-4
0.025	-3	0	-5	680.700	0	1	1
-261.138	0	1	2	34.614	0	1	3
4.055	0	1	4	-2.654	0	1	5
1.189	0	1	6	190.484	0	2	1
-164.537	0	2	2	45.432	0	2	3
-14.203	0	2	4	0.804	0	2	5
-0.709	0	2	6	68.790	0	3	1
-35.013	0	3	2	34.261	0	3	3
-10.789	0	3	4	-1.356	0	3	5
2.158	0	3	6	17.272	0	4	1
-19.791	0	4	2	9.486	0	4	3
-7.399	0	4	4	3.690	0	4	5
-0.447	0	4	6	4.167	0	5	1
-10.080	0	5	2	5.357	0	5	3
-3.124	0	5	4	0.434	0	5	5
0.148	0	5	6	0.857	0	6	1
0.043	0	6	2	2.589	0	6	3
-0.690	0	6	4	2.452	0	6	5
-0.243	0	6	6	-418.857	0	-1	-1
160.849	0	-1	-2	-45.538	0	-1	-3
4.054	0	-1	-4	-1.898	0	-1	-5
-163.029	0	-2	-1	146.128	0	-2	-2
-44.438	0	-2	-3	14.619	0	-2	-4
-5.373	0	-2	-5	-61.894	0	-3	-1

48.106	0	-3	-2	-38.095	0	-3	-3
17.194	0	-3	-4	-6.252	0	-3	-5
-12.497	0	-4	-1	14.386	0	-4	-2
-8.728	0	-4	-3	6.124	0	-4	-4
-3.965	0	-4	-5	-5.636	0	-5	-1
12.173	0	-5	-2	-6.595	0	-5	-3
4.333	0	-5	-4	-2.215	0	-5	-5
2.930	3	1	1	-3.514	3	1	2
-0.288	3	1	3	0.054	3	1	4
0.967	3	1	5	-0.417	3	1	6
-13.779	3	2	1	2.171	3	2	2
-1.952	3	2	3	0.327	3	2	4
-0.588	3	2	5	0.393	3	2	6
21.586	3	3	1	-5.047	3	3	2
5.948	3	3	3	-2.249	3	3	4
0.307	3	3	5	-0.352	3	3	6
-9.384	3	4	1	3.161	3	4	2
-7.366	3	4	3	2.164	3	4	4
0.041	3	4	5	0.071	3	4	6
5.648	3	5	1	-8.949	3	5	2
7.936	3	5	3	-3.338	3	5	4
1.530	3	5	5	-0.386	3	5	6
-0.846	3	6	1	2.319	3	6	2
-2.325	3	6	3	1.128	3	6	4
-0.595	3	6	5	0.106	3	6	6
-1.343	6	1	1	1.312	6	1	2
-0.403	6	1	3	0.571	6	1	4
-0.565	6	1	5	0.220	6	1	6
2.074	6	2	1	-1.320	6	2	2
0.620	6	2	3	-0.728	6	2	4
0.489	6	2	5	-0.368	6	2	6
-2.672	6	3	1	1.577	6	3	2
-1.212	6	3	3	0.909	6	3	4
-0.512	6	3	5	0.344	6	3	6
1.812	6	4	1	-0.877	6	4	2
1.376	6	4	3	-0.716	6	4	4
0.656	6	4	5	-0.317	6	4	6
-1.630	6	5	1	0.806	6	5	2
-1.320	6	5	3	0.627	6	5	4
-0.792	6	5	5	0.216	6	5	6
0.701	6	6	1	-0.138	6	6	2
0.606	6	6	3	-0.201	6	6	4
0.281	6	6	5	-0.149	6	6	6
0.642	3	-1	-1	1.452	3	-1	-2
0.464	3	-1	-3	-0.369	3	-1	-4
0.231	3	-1	-5	9.164	3	-2	-1
-1.576	3	-2	-2	1.176	3	-2	-3
0.107	3	-2	-4	-0.374	3	-2	-5
-20.315	3	-3	-1	7.233	3	-3	-2
-5.114	3	-3	-3	0.452	3	-3	-4
0.634	3	-3	-5	10.131	3	-4	-1
-5.387	3	-4	-2	5.763	3	-4	-3
-0.724	3	-4	-4	-0.500	3	-4	-5
-4.302	3	-5	-1	6.770	3	-5	-2
-4.602	3	-5	-3	0.966	3	-5	-4
0.296	3	-5	-5	0.328	6	-1	-1
-0.357	6	-1	-2	-0.065	6	-1	-3
0.142	6	-1	-4	-0.036	6	-1	-5

-1.301	6	-2	-1	0.488	6	-2	-2
-0.304	6	-2	-3	0.188	6	-2	-4
-0.008	6	-2	-5	1.489	6	-3	-1
-0.915	6	-3	-2	0.331	6	-3	-3
0.178	6	-3	-4	0.047	6	-3	-5
-1.167	6	-4	-1	0.155	6	-4	-2
-0.366	6	-4	-3	-0.044	6	-4	-4
-0.090	6	-4	-5	0.361	6	-5	-1
0.064	6	-5	-2	0.078	6	-5	-3
-0.030	6	-5	-4	0.050	6	-5	-5
-8.415	-3	-1	1	6.474	-3	-1	2
-0.003	-3	-1	3	-0.618	-3	-1	4
0.224	-3	-1	5	0.176	-3	-1	6
14.078	-3	-2	1	-0.940	-3	-2	2
1.146	-3	-2	3	0.274	-3	-2	4
-0.086	-3	-2	5	0.092	-3	-2	6
-22.540	-3	-3	1	5.030	-3	-3	2
-6.101	-3	-3	3	1.240	-3	-3	4
0.580	-3	-3	5	-0.105	-3	-3	6
7.201	-3	-4	1	-1.867	-3	-4	2
6.959	-3	-4	3	-1.499	-3	-4	4
-0.631	-3	-4	5	0.342	-3	-4	6
-3.779	-3	-5	1	6.528	-3	-5	2
-4.959	-3	-5	3	1.213	-3	-5	4
0.132	-3	-5	5	-0.178	-3	-5	6
-0.274	-3	1	-1	0.472	-3	1	-2
-0.166	-3	1	-3	0.096	-3	1	-4
-0.139	-3	1	-5	11.354	-3	2	-1
-1.479	-3	2	-2	2.096	-3	2	-3
0.107	-3	2	-4	-0.497	-3	2	-5
-20.910	-3	3	-1	7.866	-3	3	-2
-5.584	-3	3	-3	0.131	-3	3	-4
0.875	-3	3	-5	9.266	-3	4	-1
-4.451	-3	4	-2	5.920	-3	4	-3
-0.978	-3	4	-4	-0.401	-3	4	-5
-4.598	-3	5	-1	9.132	-3	5	-2
-5.772	-3	5	-3	2.156	-3	5	-4
-0.191	-3	5	-5	0.154	-3	6	-1
-1.817	-3	6	-2	1.237	-3	6	-3
-0.387	-3	6	-4	0.126	-3	6	-5
